# Suitability of current typing procedures to identify epidemiologically linked human *Giardia duodenalis* isolates

**DOI:** 10.1371/journal.pntd.0009277

**Published:** 2021-03-25

**Authors:** Andreas Woschke, Mirko Faber, Klaus Stark, Martha Holtfreter, Frank Mockenhaupt, Joachim Richter, Thomas Regnath, Ingo Sobottka, Ingrid Reiter-Owona, Andreas Diefenbach, Petra Gosten-Heinrich, Johannes Friesen, Ralf Ignatius, Toni Aebischer, Christian Klotz

**Affiliations:** 1 Department of Infectious Diseases, Unit for Mycotic and Parasitic Agents and Mycobacteria, Robert Koch Institute, Berlin, Germany; 2 Laboratory of Innate Immunity, Institute of Microbiology, Infectious Diseases and Immunology, Charité - Universitätsmedizin Berlin, Corporate Member of Freie Universität Berlin, Humboldt-Universität zu Berlin, and Berlin Institute of Health, Campus Benjamin Franklin, Berlin, Germany; 3 Department for Infectious Disease Epidemiology, Gastrointestinal Infections, Zoonoses and Tropical Infections Unit, Robert Koch Institute, Berlin, Germany; 4 Department of Gastroenterology, Hepatology and Infectiology, University Hospital Düsseldorf, Düsseldorf, Germany; 5 Institute of Tropical Medicine and International Health, Charité University Medicine and Berlin Institute of Health, Corporate member of Free University Berlin and Humboldt University Berlin, Berlin, Germany; 6 Laboratory Enders and Partners, Stuttgart, Germany; 7 LADR GmbH, Medizinisches Versorgungszentrum, Geesthacht, Germany; 8 Institute of Medical Microbiology, Immunology and Parasitology (IMMIP), University Clinic Bonn, Germany; 9 Department of Microbiology and Hygiene, Labor Berlin, Charité - Vivantes GmbH, Berlin, Germany; 10 MVZ Labor 28, Berlin, Germany; Universidade Federal de São Paulo, BRAZIL

## Abstract

**Background:**

*Giardia duodenalis* is a leading cause of gastroenteritis worldwide. Humans are mainly infected by two different subtypes, i.e., assemblage A and B. Genotyping is hampered by allelic sequence heterozygosity (ASH) mainly in assemblage B, and by occurrence of mixed infections. Here we assessed the suitability of current genotyping protocols of *G*. *duodenalis* for epidemiological applications such as molecular tracing of transmission chains.

**Methodology/Principal findings:**

Two *G*. *duodenalis* isolate collections, from an outpatient tropical medicine clinic and from several primary care laboratories, were characterized by assemblage-specific qPCR (*TIF*, *CATH* gene loci) and a common multi locus sequence typing (MLST; *TPI*, *BG*, *GDH* gene loci). Assemblage A isolates were further typed at additional loci (*HCMP22547*, *CID1*, *RHP26*, *HCMP6372*, *DIS3*, *NEK15411*).

Of 175/202 (86.6%) patients the *G*. *duodenalis* assemblage could be identified: Assemblages A 25/175 (14.3%), B 115/175 (65.7%) and A+B mixed 35/175 (20.0%). By incorporating allelic sequence heterozygosity in the analysis, the three marker MLST correctly identified 6/9 (66,7%) and 4/5 (80.0%) consecutive samples from chronic assemblage B infections in the two collections, respectively, and identified a cluster of five independent patients carrying assemblage B parasites of identical MLST type. Extended MLST for assemblage A altogether identified 5/6 (83,3%) consecutive samples from chronic assemblage A infections and 15 novel genotypes. Based on the observed A+B mixed infections it is estimated that only 75% and 50% of assemblage A or B only cases represent single strain infections, respectively. We demonstrate that typing results are consistent with this prediction.

**Conclusions/Significance:**

Typing of assemblage A and B isolates with resolution for epidemiological applications is possible but requires separate genotyping protocols. The high frequency of multiple infections and their impact on typing results are findings with immediate consequences for result interpretation in this field.

## Author summary

*Giardia duodenalis* is a leading cause of gastroenteritis worldwide. Humans are mainly infected by the two different genetic subtypes, assemblage A and B. Molecular typing tools for epidemiological applications such as tracking transmission, attribution to a source and outbreak investigations have been developed and are highly desirable. However, to what degree the tetraploid genome with allelic sequence heterogeneity (ASH), and the frequent occurrence of mixed, assemblage A and B infections hamper performance is unclear. Here, we assessed the suitability of current genotyping protocols for deciphering the molecular epidemiology of *G*. *duodenalis*. Against a common reporting bias, we incorporated ASH in the analysis and we show that typing with resolution for epidemiological applications is possible for both, assemblage A and B isolates, but requires separate protocols. We also demonstrate how the high frequency of multiple infections overall impacts on typing results, which has immediate consequences for result interpretation in this field.

## Introduction

The protozoan parasite *Giardia duodenalis* (syn. *G*. *intestinalis*, *G*. *lamblia*) is a leading cause of diarrheal disease and a relevant public health problem [1]. Laboratory diagnostics of *G*. *duodenalis* are well established, but these methods are inadequate for parasite genotyping, thus molecular epidemiological tracing of transmission chains, source attribution and outbreak investigations are hindered [1–3]. Current diagnostics cannot discriminate the eight genetically distinguishable assemblages within the *G*. *duodenalis* species complex, with assemblages A and B being the major two of these causing human disease [1]. In rare cases, assemblage E that is usually found in ruminants has also been associated with human disease [1]. Importantly, due to the lack of adequate *in vitro* culture systems genotyping analyses are mainly performed on parasite cysts in faecal samples by bulk DNA preparation of enriched cysts or of total stool samples. Such DNA samples are often referred to as isolates [4]. Interpretation of genotyping results is highly complex for several reasons: *Giardia* parasites are binucleated and possess tetraploid genomes with different grades of allelic sequence heterozygosity (ASH). In assemblage B isolates, ASH is typically greater than in assemblage A isolates. ASH per bp is estimated at 0.01–0.04% for assemblage A [5,6] and is nearly tenfold higher (at 0.5%) for assemblage B [5,7,8]. Therefore, different strategies for molecular typing of assemblage A and B are required to achieve the same power of resolution [3,9]. Moreover, when typing DNA of an isolate as defined above, distinguishing monotypic infections with a strain expressing high ASH and infections with multiple strains exhibiting low ASH becomes a formidable task. Single cyst analysis to circumvent the pitfalls due to multiple infection is possible [10] but not practically feasible, in particular not for multi-locus sequence typing (MLST) approaches. Thus, a reliable tool to characterize isolates for outbreak and source attribution will rely on DNA-based procedures on cysts directly derived from stool samples [11]. The aim of this study was to evaluate and qualify genotyping protocols that could be used for epidemiological applications such as molecular tracing of transmission chains, source attribution and outbreak investigations.

## Methods

### Ethics statement

This study was approved by the ethical review committee of the Charité-University Medicine Berlin (EA4/171/19). Under the Protection against Infection Act, Giardiasis is a notifiable disease in Germany and the study conducted in accordance to the Protection against Infection Act’s §13. In this context, informed consent was not required.

### Sample collection

The first set of samples was collected 2012–2019 at one outpatient tropical medicine clinic predominantly treating returning travellers. The collection comprised of 112 samples from 64 patients, including 24 chronically infected patients who provided more than one stool sample (longitudinal cases). Longitudinal cases served as a test-set to evaluate the power of typing procedures to identify epidemiologically linked cases.

The second set of samples (n = 171 from 138 patients, including 23 longitudinal cases) was collected 2017 through 2019 at non-hospital associated primary care laboratories. It represented a broader patient cohort, including persons without travel history. This collection was used to further evaluate the typing procedure and to investigate possible molecular differences between autochthonous and travel-associated cases.

Both sets of samples derived from patients who had been tested for *Giardia* because of gastrointestinal symptoms.

### Molecular and sequence analysis

The workflow of the molecular analysis is depicted in Supplementary S1 Fig. All procedures were performed according to published protocols and information of primer sequences and accession numbers of reference sequences are provided in supplementary tables (S1, S2 and S3 Tables).

#### DNA extraction

*Giardia* positive stool samples were sent from primary diagnostic laboratories to the Robert Koch-Institute, were stored at 4°C and processed within two weeks. *Giardia* cysts were enriched from stool samples by sucrose gradient flotation as previously described [12] and DNA extraction was performed using the Maxwell 16 FFPE Plus LEV DNA Purification Kit (Promega Corporation, Wisconsin, USA) following the manufacture´s protocol. DNA from samples with lower cyst counts (≤ 10^5^ cysts/g faeces) was directly extracted from stool using the QIAamp Fast DNA Stool Mini Kit (Qiagen, Hilden, Germany) according to the manufacturer´s instructions. DNA of axenically cultured trophozoites was derived from an in-house biobank (isolate 347–02, 350–01 and GS (ATCC 50581)). The principle workflow to establish axenic cultures is described elsewhere [12].

#### Real-time PCR assays to assess assemblage type

All samples were screened for the presence of assemblage A and B infections by real-time PCR to assess assemblage type. Therefore, a 168-bp amplicon at the *TIF* locus for detection assemblage A and a 99-bp fragment of the *CATH* gene for identification of assemblage B was detected according to a previously described protocol [13]. Reactions were performed in duplicates using Maxima SYBR Green/ROX qPCR Master Mix (Thermo Scientific) and 1–2 μl target DNA. The PCR with *TIF* and *CATH* specific primer were done separately, respectively. Detection and data analysis were done using CFX96 Touch Real-Time PCR Detection System and CFX Maestro 1.1 software (Bio-Rad). Samples were considered positive when melting peaks were at 80.5–82.0°C for assemblage A and 75.5–78.0°C for assemblage B, respectively [13].

**MLST at *TPI*, *BG* and *GDH* loci (common typing scheme)** were performed by nested-PCR approaches and Sanger sequencing according to published protocols [14–16]. All reactions were carried out using DreamTaq DNA polymerase system (Thermo Scientific) and 1–2 μl of sample DNA for the primary PCR and 1–2.5 μl of the first PCR product for the nested PCR. *G*. *duodenalis* positive DNA and nuclease-free water were used as positive and negative controls in each run. The primer sequences were omitted from the sequences for the final sequence analyses and resulted in a *TPI* fragment of 490 bp, a *BG* fragment of 475 bp and *GDH* fragment of 393 bp. Sequences were concatenated (*TPI-BG-GDH*) for analysis resulting in a 1358 bp fragment (see also sequence analysis below).

**MLST of assemblage A isolates** were performed by nested-PCR and Sanger sequencing according a previously published protocol at the gene loci *HCMP22547*, *CID1*, *RHP26*, *HCMP6372*, *DIS3* and *NEK15411* [3]. Final fragment size for analysis was 555 bp for *HCMP22547*, 534 bp for *CID1*, 513 bp for *RHP26*, 564 bp for *HCMP6372*, 615 bp for *DIS3* and 633 bp for *NEK15411*. PCR was performed using the DreamTaq DNA polymerase system (Thermo Scientific) and 1–2μl of sample DNA for the primary PCR and 1–2.5 μl of the first PCR product for the nested PCR. *G*. *duodenalis* positive DNA and nuclease-free water were used as positive and negative controls in each run. Sequences were concatenated (order: *HCMP22547*, *CID1*, *RHP26*, *HCMP6372*, *DIS3* and *NEK15411*) for analysis resulting in a 3414 bp fragment (see also sequence analysis below).

#### DNA sequencing and sequence analysis

Amplified DNA was purified with Exo Sap-IT PCR Product Cleanup (Thermo Scientific) according to the manufacture´s protocol. All PCR amplicons were sequenced by Sanger sequencing in both directions in an in-house facility of the RKI. Sequences were analysed using the implemented analysis tools in the software package Geneious 11.1.5 (Biomatters). Briefly, chromatograms of raw reads were all inspected visually and annotated manually if necessary to adequately annotate heterozygous positions. Ambiguous positions were evaluated using the “heterozygotes plugin” of the software tool and based on the height of the double peaks. At least 25% peak high of the lower peak was defined as cut-off for a heterozygote position and was only counted if present in both sequencing directions. Multiple alignments with reference strains were created to determine assemblages and sub-assemblages of *G*. *duodenalis* isolates. Depending on MLST scheme, respective sequences of one sample were concatenated and aligned. Distance matrix was retrieved from the alignment and implemented in GraphPad Prim (GraphPad software, LLC) for calculation and visualization. Additional information about reference sequences is provided in S2 and S3 Tables.

#### Definition of related isolates

To identify potentially linked isolates we determined the mean number and standard deviation of SNP differences of each isolate to all other isolates of the respective dataset. Two isolates were determined as potentially related if they reciprocally reached the cut-off of mean minus two standard deviations, respectively (see example in S2 Fig).

#### Phylogenetic analysis

Phylogenetic analyses were performed using MEGA 7.0.26 software [17]. Sequence data of *TPI*, *BG* and *GDH* or *HCMP22547*, *CID1*, *RHP26*, *HCMP6372*, *DIS3* and *NEK15411* genes were concatenated and Neighbor-Joining method based on the maximum composite likelihood model with pairwise deletions was applied. Bootstrap analysis using 1000 replicates was performed to review the trees reliability and values above 50% were reported. The trees were depicted unrooted.

#### Statistical analysis

Results of molecular typing were linked to the respective entry among laboratory-confirmed giardiasis cases notified to the RKI according to the infection protection act. This was done to obtain the likely country of infection according to the travel history of the patient and to identify/verify whether the developed method is sensitive and specific enough to detect consecutive samples of one patient. Statistical analysis was performed using Graph Pad Prism 8.4.0 software. Fisher´s exact test was applied and P values ≤ 0.05 were considered significant. Maps were created using the Free and Open Source Software QGIS and free vector data available at naturalearthdata.com. Estimating the likelihood of infection with single, double, and multiple strains was done using geometric progression model. In case of detection of an assemblage A or B genotype only, this can be modelled on a geometric progression of the term $\sum_{k=0}^{\infty }{\left(P(monoAss)\right)}^{k+1}$ where P(monoAss) refers to the probability of single strain infection and for which the observed frequencies with which assemblage B (B plus mixed B/A) or A (A plus B/A) were detected provided estimates of the resulting sum.

#### Nucleotide sequence accession numbers

Nucleotide sequences generated in this study have been deposited into the GenBank database (accession numbers MT878631—MT879098).

## Results

### Assignment of assemblage A versus B infections as a way to estimate likelihood of mono- and multi-strain infections

In 175 of 202 (86.6%) patients, the assemblage type could be determined (S1 Fig). Patients were predominantly infected with assemblage B (n = 115/175, 65.7%, Table 1). Co-infections with A and B were seen in 35/175 patients (20.0%). There was no statistically significant difference between patients infected with assemblages A, B or mixed infection regarding age, sex or importation status (autochthonous vs. travel associated) (chi-square: p>0.3). Among patients who likely acquired the infection abroad (n = 102, mean age: 37.8 years; 54.9% women), the specific country of infection was known for n = 93 (S3 Fig).

**Table 1:** Number (%) of patients with successful assemblage typing results by detected assemblage (A, B and A+B mixed infection), age group, sex and likely place of infection (n = 175).

	*Assemblage A* *n (%)*	*Assemblage B* *n (%)*	*Ass. A/B mixed infection* *n (%)*	*Total* *n (%)*
**Age group (years)**				
0–19	5 (20%)	18 (16%)	4 (11%)	27 (15%)
20–39	8 (32%)	44 (38%)	13 (37%)	65 (37%)
40–59	5 (20%)	38 (33%)	15 (43%)	58 (33%)
60–99	7 (28%)	14 (12%)	3 (9%)	24 (14%)
Unknown	0 (0%)	1 (1%)	0 (0%)	1 (1%)
**Sex**				
male	13 (52%)	69 (60%)	15 (43%)	97 (55)
**Likely place of infection**				
Germany	12 (48%)	39 (34%)	11 (31%)	62 (35%)
Other country	10 (40%)	70 (61%)	22 (63%)	102 (58%)
Unknown	3 (12%)	6 (5%)	2 (6%)	11 (6%)
**Total**	25 (100%)	115 (100%)	35 (100%)	175 (100%)

The data allowed estimating the likelihood of infection with single, double, and multiple strains. In case of detection of an assemblage A or B genotype only, this was modelled using a geometric progression term. Accordingly, approximately 75% assemblage A type infections and 50% B type infections, respectively, were likely to be single strain infections.

### Evaluation of current MLST genotyping schemes

**Fig 1:**
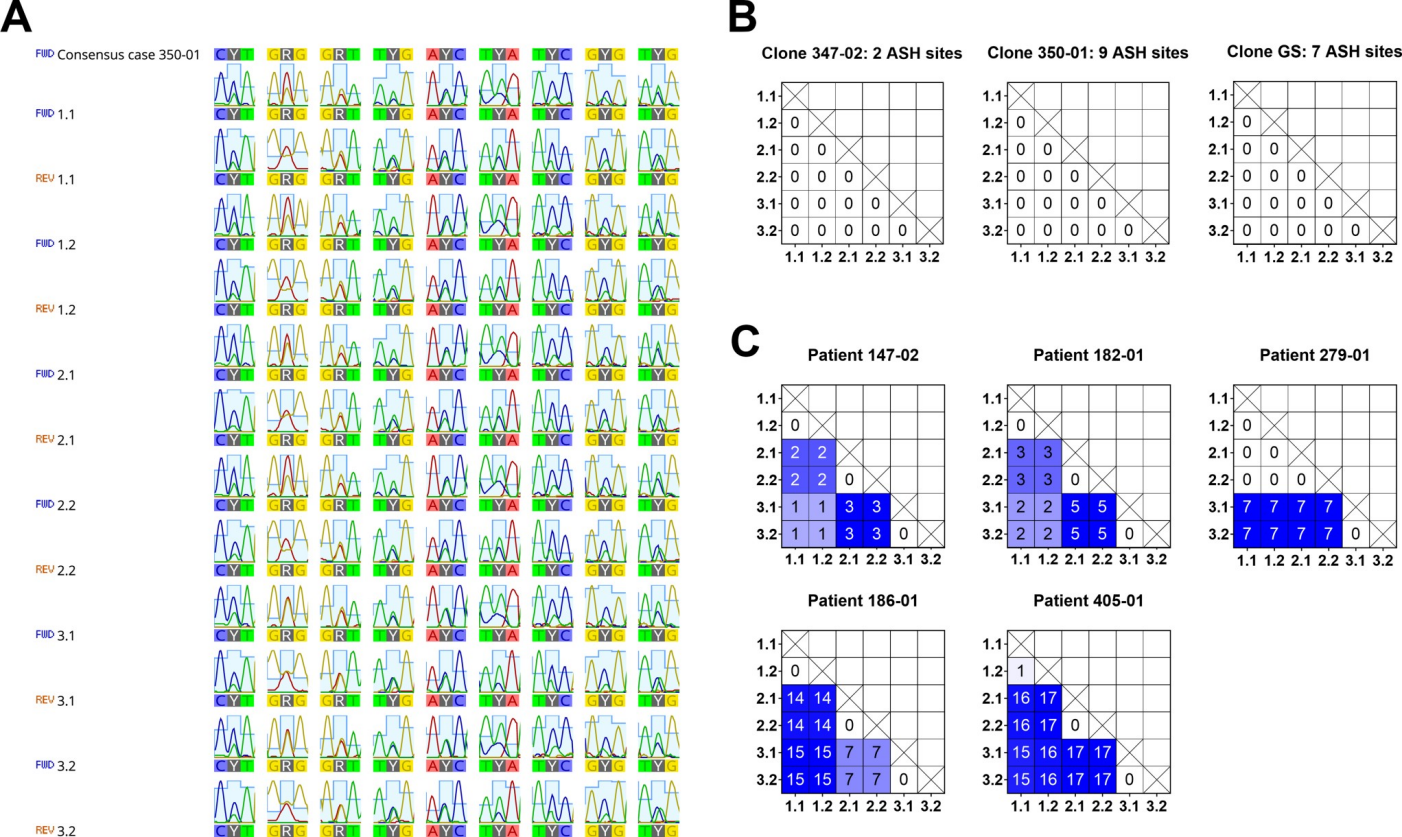
Reproducibility of ASH calling in assemblage B parasites using a common three locus typing scheme at *TPI*, *BG*, *GDH*. Each sample was typed three times in independent PCR reactions and the sequence of each PCR product was determined twice in both directions by Sanger sequencing. Results of concatenated sequences (*TPI-BG-GDH*, 1358 bp) are presented as distance matrix showing the number of pairwise differences in single nucleotide positions. Labeling depicts PCR repeats (first digit) and sequencing repeat (second digit). (A) Example depicting the chromatogram of all nine ASH sites of isolate 350–01 in multiple PCRs and after bidirectional sequencing showing the reliable identification of ASH sites in assemblage B. (B) Distance matrices of multiple PCRs showing pairwise differences in single nucleotide positions within three clonal assemblage B isolates from *in vitro* culture. Note, all ASH sites were correctly identified. (C) Distance matrices of multiple PCRs showing pairwise differences in single nucleotide positions within five assemblage B isolates derived from stool samples. Note, repeated PCRs resulted in varying apparent differences in single nucleotide positions within the same isolate.

To adapt a common protocol detecting *TPI*, *BG*, *GDH* [4], we typed cloned assemblage B strains (i.e., trophozoites from *in vitro* cultures) and selected recent assemblage B isolates (i.e., cysts enriched from stool samples). ASH in the 1358 bp long sequence covered by the three typing gene-specific PCRs was required for the validation set of templates (Fig 1). Evaluation consisted of comparison of three independent fragments generated from the same template DNA, in which every fragment was sequenced twice (Fig 1). Repeated typing of cloned parasite strains reproduced to 100% the MLST type, which included ASH residues indicating that our workflow was robust (Fig 1A and 1B). Repeated typing of assemblage B isolates surprisingly produced different and distinct degrees of sequence variation between independently generated typing fragments while sequencing/resequencing pairs of the respective fragments was except for one occasion always identical (Fig 1C).

**Fig 2:**
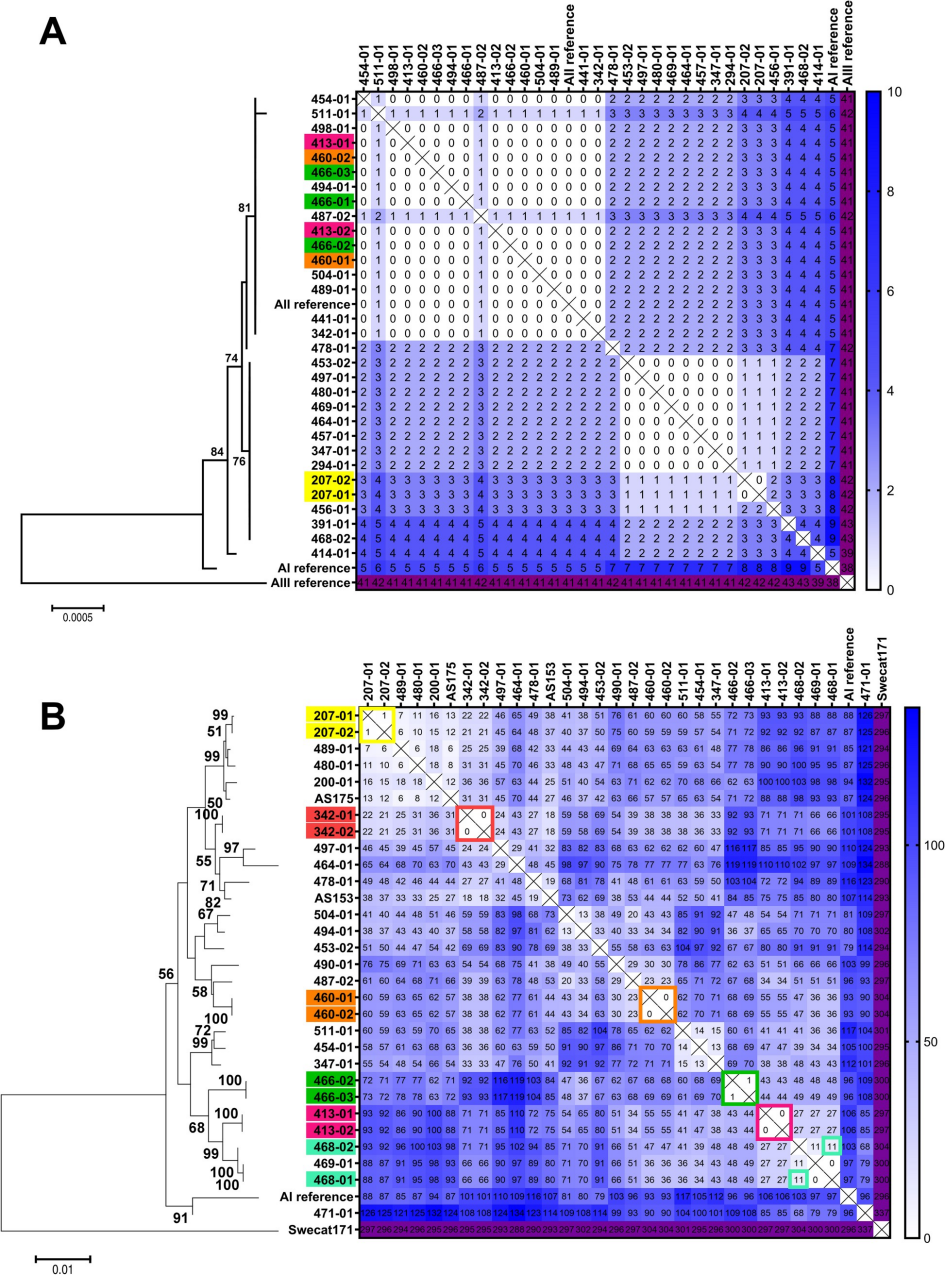
Distance matrices of pairwise comparison of *G*. *duodenalis* assemblage A isolates. (A) Results of concatenated sequences of common typing scheme (*TPI-BG-GDH*, 1358 bp) are presented as distance matrix showing the number of pairwise differences in single nucleotide positions. Phylogram of neighbour-joining analysis (only bootstrap values above 50 are shown) is included for illustration. (B) Sequence comparison of concatenated sequences of assemblage A specific typing scheme (*HCMP22547-CID1-RHP26-HCMP6372-DIS3-NEK15411*, 3414 bp) is presented as distance matrix showing the number of pairwise differences in single nucleotide positions. Phylogram of neighbour-joining analysis (only bootstrap values above 50 are shown) is included for illustration. Note, isolates are not identical with (A) due to varying typing efficiency of isolates. Colored case labels highlight longitudinal samples from chronically infected patients. Scale bars denote substitutions per site.

Typing of assemblage A isolates, for which ASH is low and MLST using *TPI*, *BG*, *GDH* has very low discriminatory power, was performed to further assess the principle robustness of the protocol (Fig 2A). Very few SNPs were observed in the assemblage A isolates and differences were mostly shared between strains and not reflecting ASH sites. MLST typing of assemblage A isolates was then extended to include the typing genes recently described by Ankarklev et al [3] (Fig 2B). These genes were highly polymorphic in the population while exhibiting low number of ASH sites [3].

The MLST typing data of the latter scheme (Fig 2B and S1 Data) revealed 15 novel MLST types not reported before while three were identical to the previously described MLST type 18 (2 isolates), 30 (2 isolates) and 42 (1 isolate), respectively [3]. Moreover, in five of the 22 typed isolates, ASH was observed (S1 Data). Thus, five of 22 samples could be due to mixed infections, a number close to the estimated theoretical value of 25% for assemblage A infections (see above). Of note, isolates were clearly distinct from each other with the notable exception of isolates sampled longitudinally from patients with chronic disease (Fig 2B), confirming the data of the initial study by Ankarklev et al. showing the suitability of this typing scheme to identify epidemiologically linked cases of assemblage A infections [3].

### Genotyping of assemblage B by a common MLST scheme to identify epidemiological links between infections

We evaluated the potential of the common three loci MLST typing scheme available for assemblage B isolates [1,2,10,18] for outbreak or transmission chain analysis. This has been hampered by the high degree of ASH in assemblage B, a real feature that for sequence interpretation has usually not been included [2,4,18] and results in a bias in data repositories.

**Fig 3:**
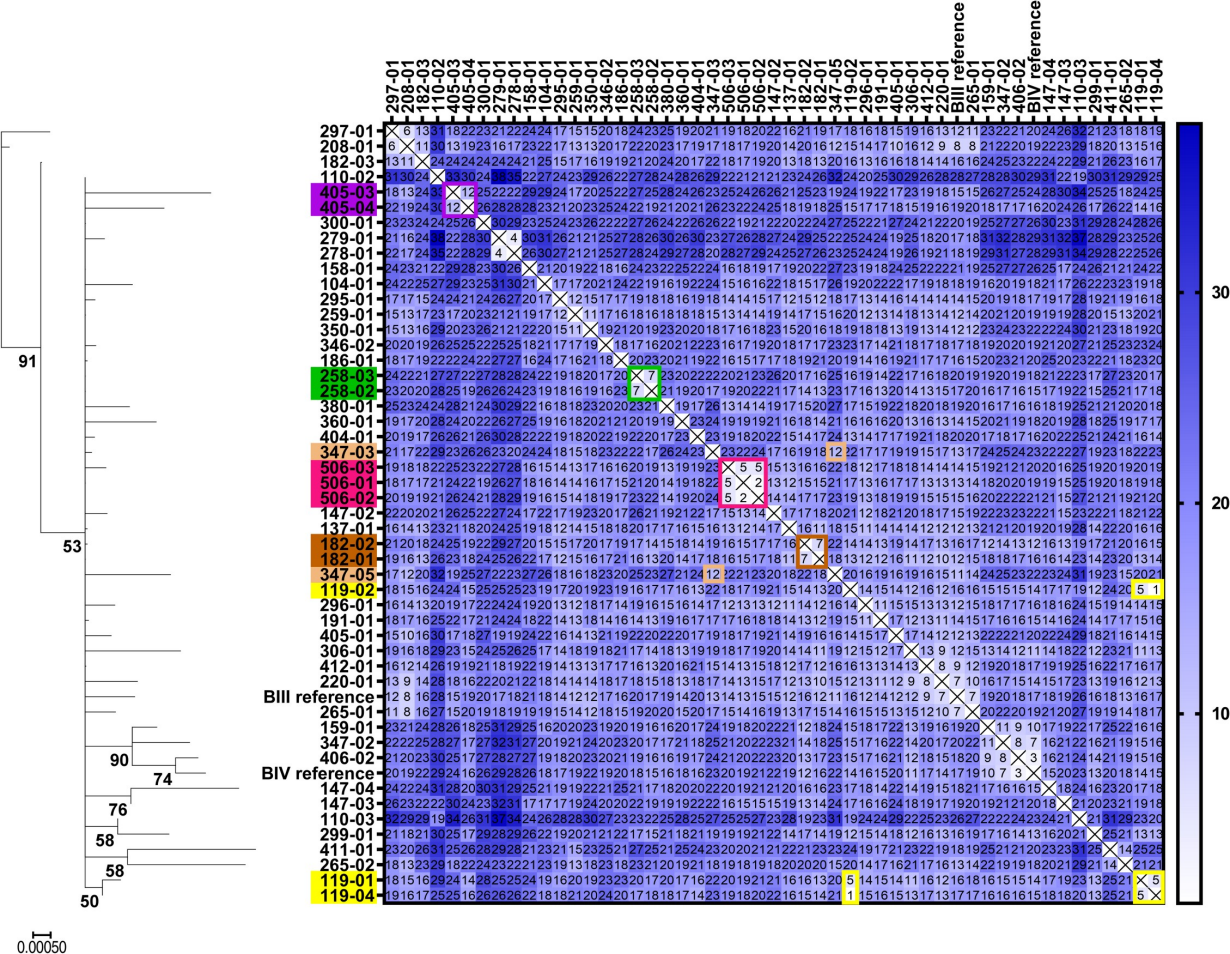
Distance matrix of pairwise comparison of *G*. *duodenalis* assemblage B isolates from a sample collection including known longitudinal samples from chronically infected patients. Comparison of concatenated sequences of a common typing scheme (*TPI-BG-GDH*, 1358 bp) for assemblage B isolates of collection 1 are presented as distance matrix showing the number of pairwise differences in single nucleotide positions. Phylogram of neighbour-joining analysis (only bootstrap values above 50 are shown) is included for illustration. Longitudinal samples from chronically infected patients were highlighted in the same colour if they met the outlier detection cut-off (mean minus 2SD). Note, six of the nine longitudinal cases were correctly identified. Three pairs of unlinked isolates also met the cut-off (cases 208–01 and 297–01, case 278–01 and 279–01, case 347–02 and 406–02). Scale bar denotes substitutions per site.

We first used a set of isolates from the first sample collection, which comprised of 49 completely typed assemblage B isolates sampled from 34 patients, including longitudinal samples of nine chronically infected cases. The latter were used as a test set for epidemiologically linked samples. The distance matrix of SNPs that were different in pairwise comparisons is shown in Fig 3. Mostly, these values were normally distributed around an average of 19.7 base sites depending on the individual isolate (see S2 Fig). Notably, pairwise testing for outliers that had fewer than the average minus 2SD differences correctly identified six of the nine longitudinal cases (Fig 3). For the three other isolate pairs typing produced a pattern consistent with multiple strain infections which as indicated before were estimated to be present in every other case of assemblage B infections. Thus, the typing algorithm with outlier detection did positively identify underlying epidemiological links with a sensitivity of 6 of 9 (67%) cases. In contrast, only three pairs of non-linked cases were falsely identified in the outlier testing, estimating a specificity of about 80% of this analysis. However, assessment of specificity of the analysis is more complex since population structure will impact which subset of genotypes has to be considered to determine relevant average distances for outlier analysis. For example, a number of isolates clustered with BIII or BIV reference sequence types and showed lower SNP to strains in these clusters (Fig 3). Hence, outlier detection has to be performed taking into account these related genotypes and, as a consequence, significantly lower SNPs have to be observed to infer epidemiological links.

**Fig 4:**
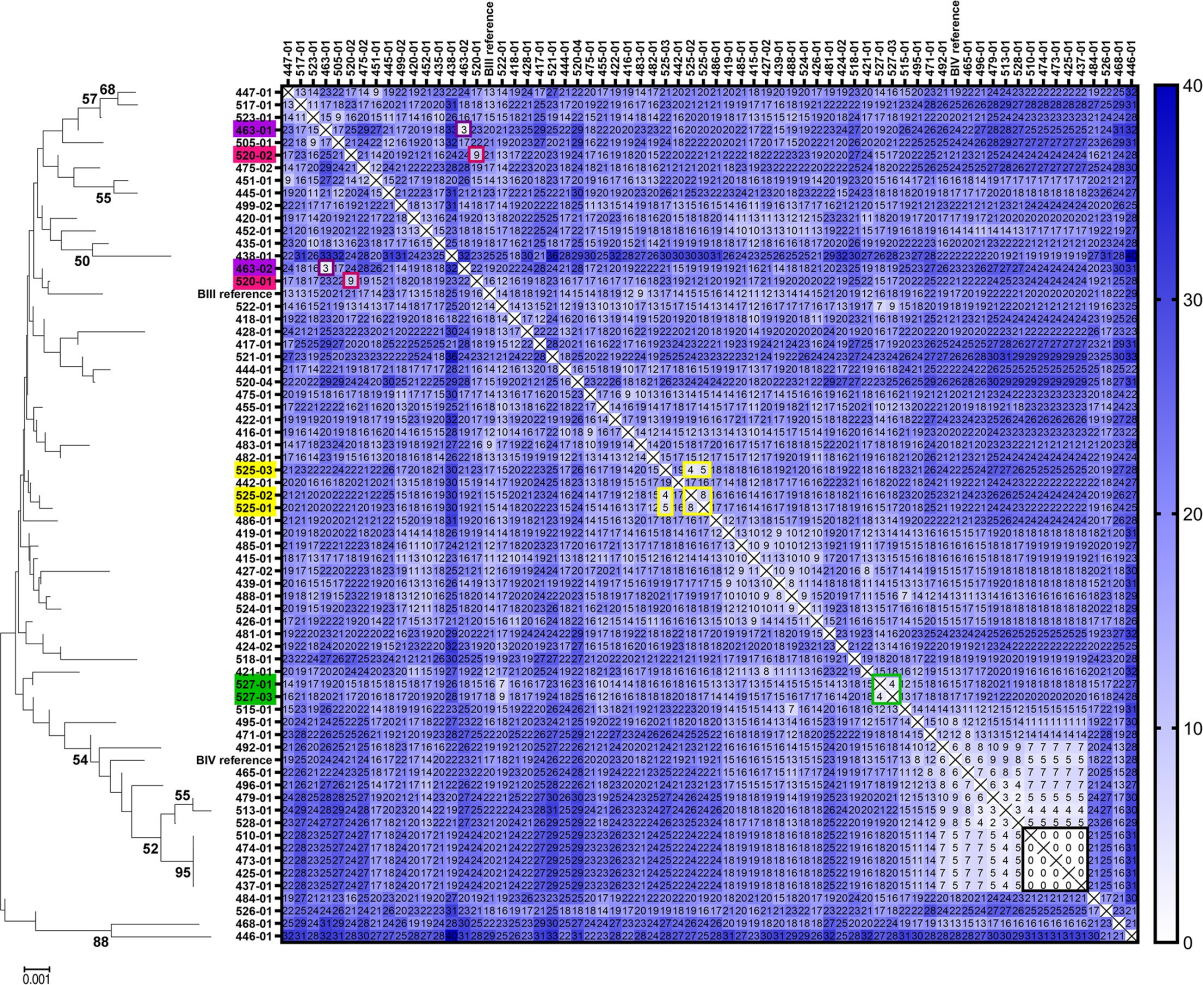
Distance matrix of pairwise comparison of *G*. *duodenalis* assemblage B isolates of consecutive samples to identify repeated sampling from chronic cases and possible occurrence of transmission chains. Comparison of concatenated sequences of a common typing scheme (*TPI-BG-GDH*, 1358 bp) for assemblage B isolates of collection 2 are presented as distance matrix showing the number of pairwise differences in single nucleotide positions. Phylogram of neighbour-joining analysis (only bootstrap values above 50 are shown) is included for illustration. Samples from chronically infected patients were highlighted in the same colour if they met the outlier detection cut-off (mean minus 2SD). Note, five samples revealed identical nucleotide composition suggesting a before unnoticed epidemiological link (black frame). Note furthermore the subsets of isolates around BIII- and BIV-references possessing lower number of variations in single nucleotide positions. Scale bar denotes substitutions per site.

Next, we applied the approach to samples of consecutive giardiasis cases of assemblage B mainly from Berlin, Germany, in order to test, whether such an MLST approach could identify repeated sampling of chronic cases as in the first sample set and occurrence of transmission chains. Similar to the results of the first sample set, we were able to identify a high proportion (four out of five; 80%) of longitudinal–i.e., epidemiologically linked—cases (Fig 4). In addition, a cluster of five cases was exceptional because MLST sequences were all identical and did not show any sites of ASH within the typed fragments. The potential index case of this cluster sampled first was a returning traveller who likely contracted infection in East Africa while the four consecutive cases sampled over the next 10 months were notified as autochthonous cases without a recent travel history. Notably, the isolates with this unique MLST type were very similar to seven other MLST types and highly related to reference BIV sequence type (Fig 4). This suggests that within BIV-like *G*. *duodenalis* there exists a highly related subpopulation. The latter “BIV-cluster” contained cases that acquired infection in various countries and may indicate the existence of a globally distributed common or dominant parasite genotype.

## Discussion

Using two instructive collections of giardiasis isolates from patients in Germany with and without relevant travel history, we show that MLST analysis can reveal epidemiological links with reasonable sensitivity and specificity, as illustrated by the identification of longitudinal samples from single patients. Our study confirms the usefulness of an extended MLST scheme to differentiate assemblage A strains and reports additional genotypes not described before. Importantly and based on assemblage typing, estimates of concurrent infections could be derived and their impact on the reproducibility of the MLST results demonstrated. Overall, samples were characterized by a dominance of assemblage B infections while no differences in age, sex or importation status (travel vs. no travel history) of infection were detectable between assemblages A, B or mixed infection case groups.

Overrepresentation of assemblage B in human infections has been reported by most studies, worldwide and also in Germany [1,2,9,19]. Other proportions of assemblage distribution may occur in specific settings such as during outbreaks or zoonotic transmission [9]. For example, a study performed in children in the Netherlands suggested that assemblage B infections are associated with anthroponotic infections and assemblage A with zoonotic transmission [20]. This is in line with a previous observation in Germany showing association of autochthonous infections with owning pets [21]. Although not statistically significant we also detected a slightly higher proportion of assemblage A in autochthonous vs. travel associated infections. Both assemblages are pathogenic in humans but there is overall inconsistent data whether or not they mediate different symptoms and whether infection sources differ [1,9,20].

Assemblage typing in the present study was successful in 86,6% of the cases. Failure to type all *Giardia* positive samples has been commonly described [22] and can likely be explained by the very high sensitivity required to amplify single copy gene targets that are mostly used for typing and that may not be reached in samples with low parasite load. Genotyping is applied more and more frequently to characterize *Giardia* populations in different hosts and from different geographic regions [1–3,9,10]. As shown here and by others [23,24] mixed infections, reduce typing reproducibility, which needs to be considered when interpreting typing results. Notably, standard Sanger sequencing, which is mostly used for *Giardia* typing procedures, is only able to reliably identify minor sequence variants with frequencies of 20% or higher [25]. Moreover, there is generally little appreciation of the pitfalls of interpreting the validity of the typing results. This is in particular true with respect to the issue of ASH in the tetraploid *Giardia* organisms. Typing entries in sequence databases are heavily biased towards sequences that do not contain polymorphic sites although the clinically predominant assemblage B parasites show extensive ASH. For example, analysis of 738 TPI sequences of assemblage B in GenBank revealed a proportion of 20.9% sequences with ambiguous nucleotide sites. In contrast, the respective proportion in our dataset was 59.1%. However, a useful typing methodology has to reflect ASH and we here show that workflows for reliable identification of ASH are feasible.

It has been highlighted that transmission chain identification needs different genotyping strategies depending on the underlying assemblage type [2,3,9]. For human assemblage A infections, we confirm that the newly developed MLST scheme based on six genome markers is reliable and useful to identify epidemiologically linked isolates [3] which supports its application in future studies.

We report MLST clusters related to the reference sub-assemblage types BIII and BIV. Our analysis illustrates that awareness of this population structure is critical for correctly interpreting typing data if the goal is to identify transmission events. Thresholds that establish epidemiological links based on number of SNPs, as proposed here, will critically depend on the relation of an isolate to relevant sub-populations, i.e. its kin.

Improvements of current typing approaches as shown here are useful and required for epidemiological investigations and studies that aim at correlating *Giardia* genotypes with infection outcome, such as clinical manifestations, or relevance for co-infections with other pathogens. These aspects cannot be adequately addressed currently. Of note, we focused on human samples only but future studies are needed to test whether the presented typing schemes are also suitable to identify possible zoonotic transmission from animal to humans.

The public health impact of typing, e.g., on outbreak strain definition, is critically dependent on the ability to communicate results unambiguously which requires a nomenclature. Such nomenclatures are established for bacteria (https://pubmlst.org/). For other protozoa like *Cryptosporidium* spp. typing nomenclature is already advanced, partly because these organisms are haploid [26]. An appropriate nomenclature for the tetraploid *G*. *duodenalis* may only be possible through defining allele types for sets of highly polymorphic marker genes. Full genotypes, i.e., calling respective sets of alleles, may then be possible via novel sequencing independent, CRISPR/Cas-mediated typing approaches [27]. Harnessing the power of such new approaches for genotyping *Giardia* again will require in-depth insight into the parasites’ population structure.

Whole genome sequencing at low cost may allow this insight as it is revolutionizing molecular surveillance of microbial pathogens, outbreak detection and source identification (see reviews for listeria [28] and salmonella [29,30]). The lack of a useful *G*. *duodenalis* reference of its pan- and accessory genome representing the population is delaying progress in this field [6]. The recent proof-of-concept for successful whole genome sequencing of *Giardia* cysts derived from faecal samples promises to change this [31,32].

In conclusion, the present study demonstrates the power and limitations of current genotyping approaches for *G*. *duodenalis*: Potential for identification of epidemiological links between *G*. *duodenalis* infections and limitations that are inherent attributes of the tetraploid nature of the parasite and of the frequency of concurrent infections with distinct *G*. *duodenalis* strains.

## Supporting information

S1 Fig Workflow of the molecular analysis and summary of typing results. (PDF)

S2 Fig Graphical illustration of cut-off value for exemplary samples. (PDF)

S3 Fig *G. duodenalis* assemblage type by country of infection. (PDF)

S1 Table Primer sequences used in the study. (PDF)

S2 Table Accession number of references used for analysis of the common MLST scheme. (PDF)

S3 Table Accession numbers of references used for analysis of MLST results for the assemblage A typing scheme. (PDF)

S1 Data Data table depicting overall typing results. (XLSX)
