# Supplementary material for: Suitability of current typing procedures to identify epidemiologically linked human Giardia duodenalis isolates
Source: PLoS Negl Trop Dis. 2021 Mar 25;15(3):e0009277. doi: 10.1371/journal.pntd.0009277 (PMC8023459; doi:10.1371/journal.pntd.0009277)
Supplement: S1 Fig — (PDF) [file pntd.0009277.s001.pdf]

Supplementary Figure 1

Workflow of the molecular analysis and typing results

Collection 1

112 samples of 64 patients, mainly returning travellers, including 24 longitudinal cases of chronic infections

Collection 2

171 samples of 138 patients, returning travellers and autochthonous cases, including 23 longitudinal cases of chronic infections

1) Assemblage specific PCR at single copy genes *TIF* and *CATH* to detect assemblage type A and B (199/283, 70.3%; CI 64.8-75.3%)

2) Common MLST typing scheme at loci *TPI* (198/283, 70.0%; CI 64.5-75.0%), *BG* (202/283, 71.4%; CI 65.9-76.3) and *GDH* (192/283, 67.8%; CI 62.2-73.0%); typing success at all 3 genes *TPI-BG-GDH* (161/283, 56.9%; CI 51.0-62.5%)

3) Assemblage A specific MLST typing scheme: Typing success at single genes: *HCMP22547 (33/69, 47.8%; CI 36.4-59.4%), CID1 (35/69, 50.7%; CI 39.2-62.2%), RHP26 (31/69, 44.9%; CI 33.8-56.6%), HCMP6372 (34/69, 49.3%; CI 37.8-60.8%), DIS3 (33/69, 47.8%; CI 36.5-59.4%)* and *NEK15411* (33/69, *47.8%; CI 36.5-59.4%); typing success* at all six loci (28/69, 40.6%; CI 29.8-52.4)

Assemblage	Collection 1 n=64	Collection 2 n=138	Autochthonous n=76	Travel-associated n=109
Assemblage A	6	19	12	10
Assemblage B	40	75	39	70
Co-infection A/B	14	21	11	22
Not typeable	4	23	14	7

Collection 1

Concatinated sequences available for distance matrix analysis:
Assemblage A* 8 samples (7 patients)
Assemblage B 49 samples (34 patients)

Collection 2

Concatinated sequences available for distance matrix analysis:
Assemblage A* 28 samples (24 patients)
Assemblage B 66 samples (59 patients)

* Includes samples of the common typing scheme and the assemblage A specific typing scheme. Note, the assemblage A specific typing scheme was also applied on samples of patients with assemblage A/B mixed infections to determine the assemblage A subtype in these samples. In the common MLST scheme 31 samples of 26 patients could be included and for the assemblage A specific scheme 28 samples of 22 patients could be included. Assemblage A samples of both collections were analyzed together.
