# Supplementary material for: Suitability of current typing procedures to identify epidemiologically linked human Giardia duodenalis isolates
Source: PLoS Negl Trop Dis. 2021 Mar 25;15(3):e0009277. doi: 10.1371/journal.pntd.0009277 (PMC8023459; doi:10.1371/journal.pntd.0009277)
Supplement: S2 Fig — (PDF) [file pntd.0009277.s002.pdf]

Supplementary Figure 2

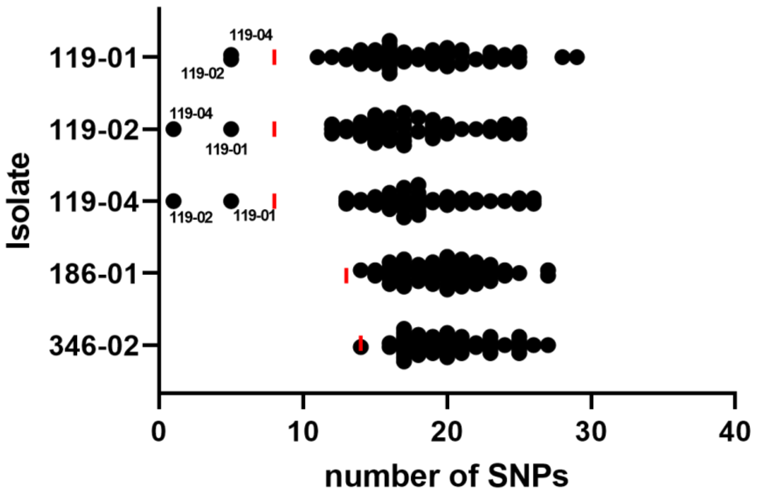

S2 Fig. Graphical illustration of cut-off value for exemplary samples. The 3 samples of longitudinal case 119 are reciprocally below the individual cut-off of mean minus 2SD (red bars). Only those samples are considered to be potentially linked that mutually reached the individual cut-off. Of all assemblage B isolates in the present study the pairwise median base sites difference of one isolate to all other isolates was 19.7 (95% confidence interval 19.2-20.2, n = 115)
