# Supplementary figures and images for: Suitability of current typing procedures to identify epidemiologically linked human Giardia duodenalis isolates
Source: PLoS Negl Trop Dis. 2021 Mar 25;15(3):e0009277. doi: 10.1371/journal.pntd.0009277 (PMC8023459; doi:10.1371/journal.pntd.0009277)

Supplementary Figure 3  
*G. duodenalis* assemblage type by country of infection

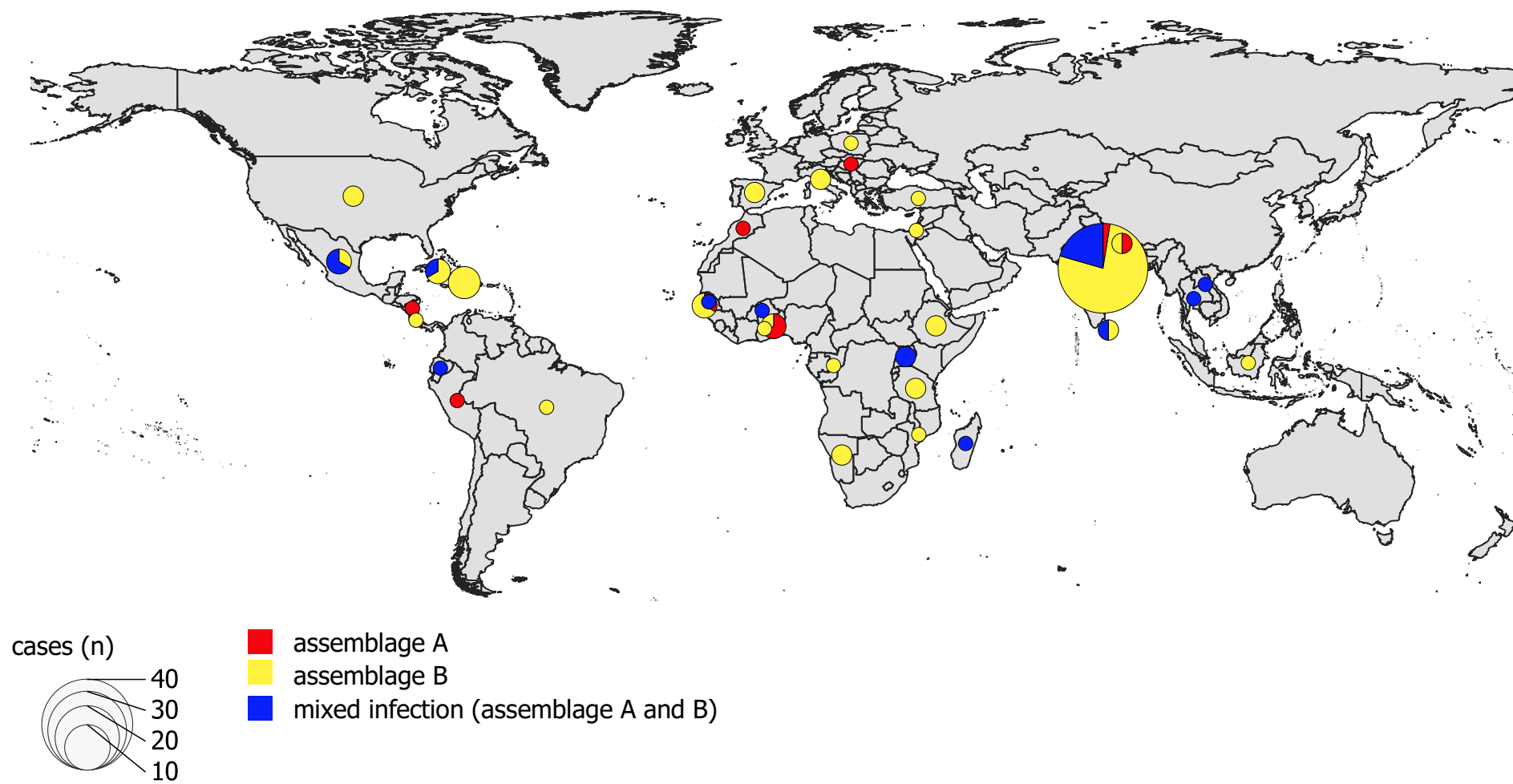

Supplement: S3 Fig — (PDF) [file pntd.0009277.s003.pdf]
