# Supplementary material for: Suitability of current typing procedures to identify epidemiologically linked human Giardia duodenalis isolates
Source: PLoS Negl Trop Dis. 2021 Mar 25;15(3):e0009277. doi: 10.1371/journal.pntd.0009277 (PMC8023459; doi:10.1371/journal.pntd.0009277)
Supplement: S1 Table — (PDF) [file pntd.0009277.s004.pdf]

S1 Table. Primer sequences used in the study.

Primer	Sequence (5´- 3´)	Fragment size (bp) PCR (fragment size (bp) used for analysis)	Reference
Translation initiation factor gene (TIF)		168	[1]
TIF_AssA-F	AGAAGTGTCCTGGACTGGGTCT		
TIF_AssA-R	CGTGGAATTGTCAATCGTTAAAC		
Cathepsin L precursor gene (CATH)		99	[1]
CATH_AssB-F	GCGATTTTCCGCGGAAGGTTGT		
CATH_AssB-R	AGAGGGCATCATAAACATAAACC		
Triosephosphate isomerase (TPI)		530 (490)	[2]
AL3543	CAAACCTTITCCGCAAACC		
AL3546	AAATIATGCCTGCTCGTCG		
AL3544	CCCTTCATCGGIGGTAACTT		
AL3545	GTGGCCACCACICCCGTGCC		
Beta-Giardin (BG)		511 (475)	[3]
G7	AAGCCCGACGACCTCACCCGCAGTGC		
G759	GAGGCCGCCCTGGATCTTCGAGACGAC		
BG-iF	GAACGAGATCGAGGTCCG		
BG-iR	CTCGACGAGCTTCGTGTT		
Glutamate dehydrogenase (GDH)		432 (393)	[4]
GDHeF	TCAACGTYAAYCGYGGYTTCCGT		
GDHiR	GTTRTCCTTGCACATCTCC		
GDHiF	CAGTACAACTCYGCTCTCGG		
GDHiR	GTTRTCCTTGCACATCTCC		
High cysteine membrane protein Group 2 (HCMP22547)		623 (555)	[5]
HCMP2 Fwd1	GACCATATTACTACGATCCAAACACG		
HCMP2 Rev1	GAGGACATGGAAGACACTTGCC		
HCMP2 Fwd2	GTCCTACGGGAGGCGTCAGT		
HCMP2 Rev2	GACTGAGGAGCGAGTAGTAACATGAT		
Caffeine-induced death protein 1-like protein (CID1)		588 (534)	[5]
CID1 Fwd1	CACGATCAGGATATGTTCCTCAGC		
CID1 Rev1	AGTGTTTATCTTCTCTTCACCTTCTGG		
CID1 Fwd2	CCATCAGTTACATCCAGAAGCACAT		
CID1 Rev2	AGTATTTGAGGCTTATCTTCAGGACG		
DNA repair and recombination protein (RHP26)		557 (513)	[5]
RHP26 Fwd1	GGTCTAGGGCTCAACCTTACTGCT		
RHP26 Rev1	CTCCAACAGCGTGTGTGTCTGTAG		
RHP26 Fwd2	GACAACGCCTCCGTCACTTC		
RHP26 Rev2	GACTCCTTGATGGCATACAACG		
High cysteine protein (HCMP6372)		640 (564)	[5]
HCMP3 Fwd1	GAACTTGTGTCACGCTGAATTAATACAG		
HCMP3 Rev1	TCGGGAACACAGACGACACCT		
HCMP3 Fwd2	GGCGGTGAGTGTGTGGAGAC		
HCMP3 Rev2	CTTCAGAGATGCAAGTACCATTGTTC		
Mitotic control protein (DIS3)		667 (615)	[5]
DIS3 Fwd1	GCAAGAAAATGAGGACATTATGCTAGAG		
DIS3 Rev1	CGTTTCTAAAATTAATGCTCCGTTTC		
DIS3 Fwd2	CCATTCCAAGCGGAGTTTATGC		
DIS3 Rev2	GCCTTAGACATATTCCTATAACCTGAGC		
NEK Kinase 15411 (NEK15411)		700 (633)	[5]
NEK kinase Fwd1	TCTCCTCAACGACCCCTCAATC		
NEK kinase Rev1	CCGAGTCTACCTCACCACACTCAC		
NEK kinase Fwd2	GATGGCAATCGGCTTCTCC		
NEK kinase Rev2	GACGAGCCTCTGCACAACATC		

References

1. Van Lith L, Soba B, Vizcaino VV, Svard S, Sprong H, Tosini F, et al. A real-time assemblage-specific PCR assay for the detection of Giardia duodenalis assemblages A, B and E in fecal samples. Vet Parasitol. 2015;211(1-2):28-34. doi: 10.1016/j.vetpar.2015.04.017. PubMed PMID: 25935292.
2. Sulaiman IM, Fayer R, Bern C, Gilman RH, Trout JM, Schantz PM, et al. Triosephosphate isomerase gene characterization and potential zoonotic transmission of Giardia duodenalis. Emerg Infect Dis. 2003;9(11):1444-52. Epub 2004/01/14. PubMed PMID: 14718089; PubMed Central PMCID: PMC3035538.
3. Lalle M, Pozio E, Capelli G, Bruschi F, Crotti D, Caccio SM. Genetic heterogeneity at the beta-giardin locus among human and animal isolates of Giardiaduodenalis and identification of potentially zoonotic subgenotypes. Int J Parasitol. 2005;35(2):207-13. Epub 2005/02/16. doi: 10.1016/j.ijpara.2004.10.022. PubMed PMID: 15710441.
4. Read CM, Monis PT, Thompson RC. Discrimination of all genotypes of Giardia duodenalis at the glutamate dehydrogenase locus using PCR-RFLP. Infect Genet Evol. 2004;4(2):125-30. Epub 2004/05/26. doi: 10.1016/j.meegid.2004.02.001. PubMed PMID: 15157630.
5. Ankarklev J, Lebbad M, Einarsson E, Franzen O, Ahola H, Troell K, et al. A novel high-resolution multilocus sequence typing of Giardia intestinalis Assemblage A isolates reveals zoonotic transmission, clonal outbreaks and recombination. Infect Genet Evol. 2018;60:7-16. doi: 10.1016/j.meegid.2018.02.012. PubMed PMID: 29438742.
