# Supplementary material for: Suitability of current typing procedures to identify epidemiologically linked human Giardia duodenalis isolates
Source: PLoS Negl Trop Dis. 2021 Mar 25;15(3):e0009277. doi: 10.1371/journal.pntd.0009277 (PMC8023459; doi:10.1371/journal.pntd.0009277)
Supplement: S2 Table — (PDF) [file pntd.0009277.s005.pdf]

S2 Table. Accession number of references used for the analysis of common multi-locus sequence typing (MLST) results [1].

| **Assemblage** | **TPI** | **BG** | **GDH** |
|---|---|---|---|
| AI | L02120 | X85958 | M84604 |
| AII | U57897 | AY072723 | AY178737 |
| AIII | EU781002 | EU769206 | EU769223 |
| BIII | AY228628 | AY072726 | AF069059 |
| BIV | L02116 | AY072725 | AY178738 |

## References

1. Sprong H, Caccio SM, van der Giessen JW, network Z, partners. Identification of zoonotic genotypes of Giardia duodenalis. PLoS Negl Trop Dis. 2009;3(12):e558. Epub 2009/12/04. doi: 10.1371/journal.pntd.0000558. PubMed PMID: 19956662; PubMed Central PMCID: PMC2777335.
