# Supplementary material for: Suitability of current typing procedures to identify epidemiologically linked human Giardia duodenalis isolates
Source: PLoS Negl Trop Dis. 2021 Mar 25;15(3):e0009277. doi: 10.1371/journal.pntd.0009277 (PMC8023459; doi:10.1371/journal.pntd.0009277)
Supplement: S3 Table — (PDF) [file pntd.0009277.s006.pdf]

S3 Table. Accession numbers of references used for analysis of MLST results for assemblage A.

Code	**CID1** Isolate/accession number	**Rhp26p** Isolate/accession number	**HCMP22547** Isolate/accession number	**HCMP6372** Isolate/accession number	**DIS3** Isolate/accession number	**NEK15411** Isolate/accession number
01	WB/ MG520215	WB/ MG520254	WB/ MG520233	WB/ MG520225	WB/ MG520263	WB/ MG520243
02	Sweh038/ MG520216	Sweh173/ MG520255	Sweh173/ MG520234	Sweh166/ MG520226	Sweh072/ MG520264	AS98/ MG520244
03	AS98/ MG520217	AS98/ MG520256	AS98/ MG520235	AS98/ MG520227	AS98/ MG520265	Sweh040/ MG520245
04	GU1116/ MG520218	AS175/ MG520257	AS175/ MG520236	AS175/ MG520228	Sweh036/ MG520266	AS175/ MG520246
05	AS175/ MG520219	Sweh152/ MG520258	Sweh166/ MG520237	Sweh063/ MG520229	AS153/ MG520267	Sweh038/ MG520247
06	Sweh099/ MG520220	Sweh038/ MG520259	Sweh078/ MG520238	Swemoose014/ MG520230	Sweh038/ MG520268	Sweh204/ MG520248
07	Sweh071/ MG520221	Swemoose014/ MG520260	Swesheep006/ MG520239	Swecat078/ MG520231	AS175/ MG520269	Swemoose014/ MG520249
08	Swesheep015/ MG520222	Swecat078/ MG520261	Swecat078/ MG520240	Swecat171/ MG520232	Swecat171/ MG520270	Sweh166/ MG520250
09	Swemoose014/ MG520223	Swecat171/ MG520262	Swecat035/ MG520241	464-01/ MT879095	464-01/ MT879093	Swesheep060/ MG520251
10	Swecat171/ MG520224	207-01/ MT879098	Swecat171/ MG520242			Swecat078/ MG520252
11			466-02/ MT879094			Swecat171/ MG520253
12						453-02/ MT879097
13						511-01/ MT879096

Yellow highlighted isolate and accession numbers have been identified in this study, while the others are published in Ankarklev et al. 2018 [1].

References

1. Ankarklev J, Lebbad M, Einarsson E, Franzen O, Ahola H, Troell K, et al. A novel high-resolution multilocus sequence typing of Giardia intestinalis Assemblage A isolates reveals zoonotic transmission, clonal outbreaks and recombination. Infect Genet Evol. 2018;60:7-16. doi: 10.1016/j.meegid.2018.02.012. PubMed PMID: 29438742.
